# TLR9 re-expression in cancer cells extends the S-phase and stabilizes p16^INK4a^ protein expression

**DOI:** 10.1038/oncsis.2016.49

**Published:** 2016-07-25

**Authors:** P Parroche, G Roblot, F Le Calvez-Kelm, I Tout, M Marotel, M Malfroy, G Durand, J McKay, M Ainouze, C Carreira, O Allatif, A Traverse-Glehen, M Mendiola, J J Pozo-Kreilinger, C Caux, M Tommasino, N Goutagny, U A Hasan

**Affiliations:** 1 CIRI, INSERM U1111, Ecole Normale Supérieure, Université de Lyon, Lyon, France; 2 IARC-International Agency for Research on Cancer 150 Cours Albert Thomas, Lyon, France; 3 CRCL, UMR INSERM 1052-CNRS 5286, Centre Léon Bérard, Lyon France; 4 Hospices Civils Lyon Sud, Pierre Benite, France; 5 Molecular Pathology and Therapeutic Targets Group, Research Insitute (IdiPAZ), La Paz University Hospital, Madrid, Spain and Molecular Pathology Diagnostics Unit, Institute of Medical and Molecular Genetics (INGEMM), La Paz University Hospital, Madrid, Spain; 6 Pathology Department, La Paz University Hospital, Madrid Spain

## Abstract

Toll-like receptor 9 (TLR9) recognizes bacterial, viral or cell damage-associated DNA, which initiates innate immune responses. We have previously shown that TLR9 expression is downregulated in several viral induced cancers including HPV16-induced cervical neoplasia. Findings supported that downregulation of TLR9 expression is involved in loss of anti-viral innate immunity allowing an efficient viral replication. Here we investigated the role of TLR9 in altering the growth of transformed epithelial cells. Re-introducing TLR9 under the control of an exogenous promoter in cervical or head and neck cancer patient-derived cells reduced cell proliferation, colony formation and prevented independent growth of cells under soft agar. Neither TLR3, 7, nor the TLR adapter protein MyD88 expression had any effect on cell proliferation, indicating that TLR9 has a unique role in controlling cell growth. The reduction of cell growth was not due to apoptosis or necrosis, yet we observed that cells expressing TLR9 were slower in entering the S-phase of the cell cycle. Microarray-based gene expression profiling analysis highlighted a strong interferon (IFN) signature in TLR9-expressing head and neck cancer cells, with an increase in IFN-type I and IL-29 expression (IFN-type III), yet neither IFN-type I nor IL-29 production was responsible for the block in cell growth. We observed that the protein half-life of p16^INK4a^ was increased in TLR9-expressing cells. Taken together, these data show for the first time that TLR9 affects the cell cycle by regulating p16^INK4a^ post-translational modifications and highlights the role of TLR9 in the events that lead to carcinogenesis.

## Introduction

Normal tissues carefully control the production and release of growth-promoting signals. These signals will allow entry and progression through the cell development and division cycle, thereby ensuring cell numbers and thus maintenance of normal tissue architecture and function. Cancer cells, by deregulating these signals, permit chronic proliferation. The G1/S checkpoint controls progression of cells through the restriction point into the DNA synthesis S-phase. The p16^INK4a^ and Kip/Cip family inhibitors control CDK activity and prevent entry into S-phase. p16^INK4a^ acts as a tumor suppressor through multiple biological functions, including the inhibition of cell cycle progression,^1^ the induction of senescence^2^ and differentiation,^3^ and its involvement in apoptosis^4^ and DNA repair.^5^ Overexpression of the *p16*^*INK4a*^ gene induced the inhibition of cell proliferation, which has mainly been considered to result from arrest in G1 phase of the cell cycle^6^ as well as the lengthening of S-phase.^7^

Toll-like receptors (TLRs) are expressed in many hematopoietic cell types, and their role in immune responses has been well documented.^8^ However, TLRs are also expressed in non-hematopoietic cells and have an important role in tissue homeostasis as well as cell proliferation.^9, 10, 11, 12, 13^ In certain cell types, TLR-dependent signaling results in apoptosis with a mechanism that, in part, depends on the production of type I interferon (IFN).^14, 15, 16^ The link between TLR signaling and cell cycle control has been addressed in our previous studies in which we found that flagellin, a TLR5 agonist, can induce cell cycle entry by overcoming p27-induced cell cycle arrest fibroblasts. Our findings also suggested that the differential capacity of TLR3 and TLR4 ligands to induce cell cycle progression is dependent on the ability of these ligands to produce IFN.^14, 17^ TLR9 was the first innate immune receptor identified to recognize unmethylated double-stranded DNA CpG motifs expressed in the genome of viruses and bacteria. TLR9 can become activated in response to endogenous double-stranded DNA motifs released as danger-associated molecular patterns (DAMPs).^18^ We and others have observed that oncoviruses such as human papillomavirus 16 and 38 (HPV16 and 38), Epstein Barr virus, Hepatitis B virus and Merkel cell virus impair the expression and function of the innate immune receptor TLR9 (1, 2, 14, 27). Furthermore, overexpression of TLR9 (with an exogenous promoter) in human keratinocytes transduced with HPV38E6E7 decreased their ability to grow.^19^ Thus, in addition to its role in innate immunity, TLR9 could control events that promote transformation of epithelial cells or cell growth by itself. Here, we describe a role for TLR9 in cell cycle regulation in viral and in non-viral-induced cancers. We observed that as well as in viral induced cancers, we demonstrated in patients with head and neck cancer (that are HPV negative) that TLR9 levels were downregulated. Re-constitution of TLR9 expression in head and neck cancer cells lengthened the S-phase of the cell cycle as well as increase p16^INK4a^ stability. TLR9 overexpression in head and neck cancer cells also prevented colony formation under soft agar. These data highlight the importance of TLR9 in controlling the events that lead to transformation.

## Results

### TLR9 expression affects the doubling population of HPV16-transformed cells

**Figure 1:**
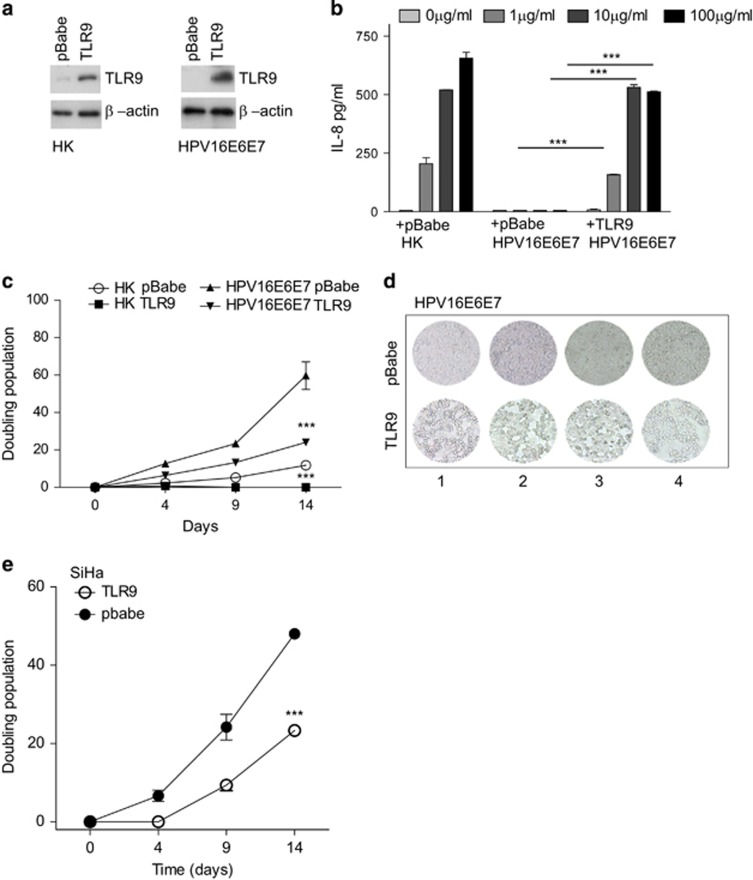
The effect of TLR9 overexpression on cell proliferation. (**a**) HK-(left panel) or HPV16E6E7-expressing keratinocytes (right panel) were stably transduced with empty or TLR9-expressing pbabe. TLR9 protein levels were determined by western blotting. (**b**) Cells were stimulated with increasing concentrations of CpG 2006 and IL-8 levels were determined by ELISA. (**c**) HK expressing or not HPV16E6E7 were stably transduced with pbabe or pbabe-TLR9 and plated for a doubling population assay. The cells were harvested and reseeded every 5 days and the doubling populations were counted. (**d**) At day 15 cells were visualized by microscopy at four different planes. (**e**) SiHa cells were stably transduced with pbabe or pbabe-TLR9 and plated for a doubling population assay. The cells were harvested and reseeded every 5 days and the doubling populations were counted. Data are representative of three independent experiments performed in triplicate. The mean±s.e.m. are shown. ***P<0.0001, based on an unpaired Student's *t*-test.

We have previously reported the ability of HPV16 viral oncoproteins E6 and E7 (HPV16E6E7) to suppress TLR9 transcription in human primary keratinocytes (HK). To determine the biological significance of HPV16-mediated downregulation of *TLR9* transcription, we re-introduced TLR9 into HK and HPV16E6E7 cells by retroviral transduction (Figure 1a). As previously reported, we observed endogenous TLR9 expression in HK but not in HPV16E6E7-transduced cells^20^ (Figure 1a). HK, but not HPV16E6E7 cells, were able to produce IL-8 when stimulated with the oligonucleotide CpG 2006, a TLR9 ligand. Yet re-expression of TLR9 restored HPV16E6E7 cells to produce IL-8 in response to CpG 2006 at levels that were comparable to HK (Figure 1b), indicating that exogenous TLR9 was functional. To determine whether TLR9 expression influenced HK or HPV16E6E7 cell growth we monitored the doubling population over a period of 14 days. HK overexpressing TLR9 failed to grow compared with HK transduced with pbabe alone. Similarly, HPV16E6E7-pbabe-TLR9 cells slowed the growth rate in comparison to HPV16E6E7-pbabe cells from 60 to 20 doublings at day 14 (Figures 1c and d). To corroborate our data, we analyzed the effect of overexpressing TLR9 (using the pbabe retroviral system) in cells derived from cervical cancer patients (SiHa) positive for HPV16. At 4, 9 and 14 days SiHa cells expressing TLR9 doubled at a slower rate than pbabe alone expressing cells (Figure 1e). Taken together, these data show that TLR9 expression prevents cell growth by reducing the doubling population of normal and HPV16-transformed cells.

### TLR9 expression is lost in head and neck cancer patients

**Figure 2:**
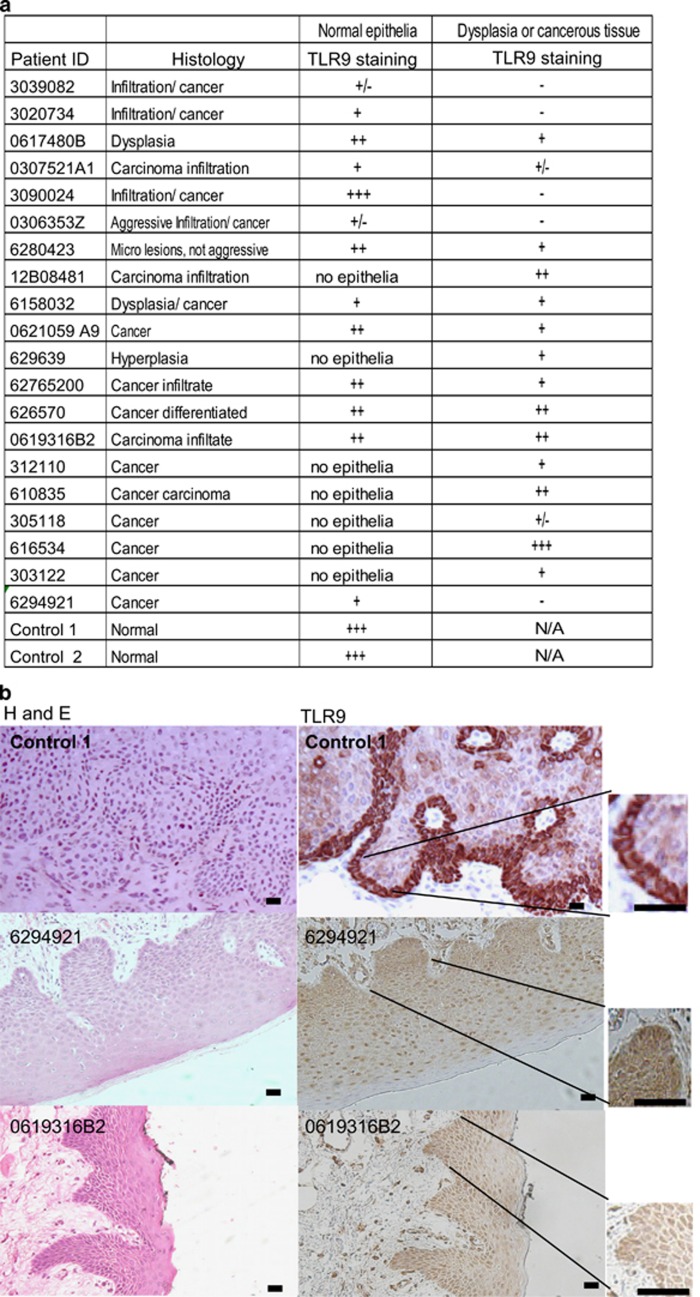

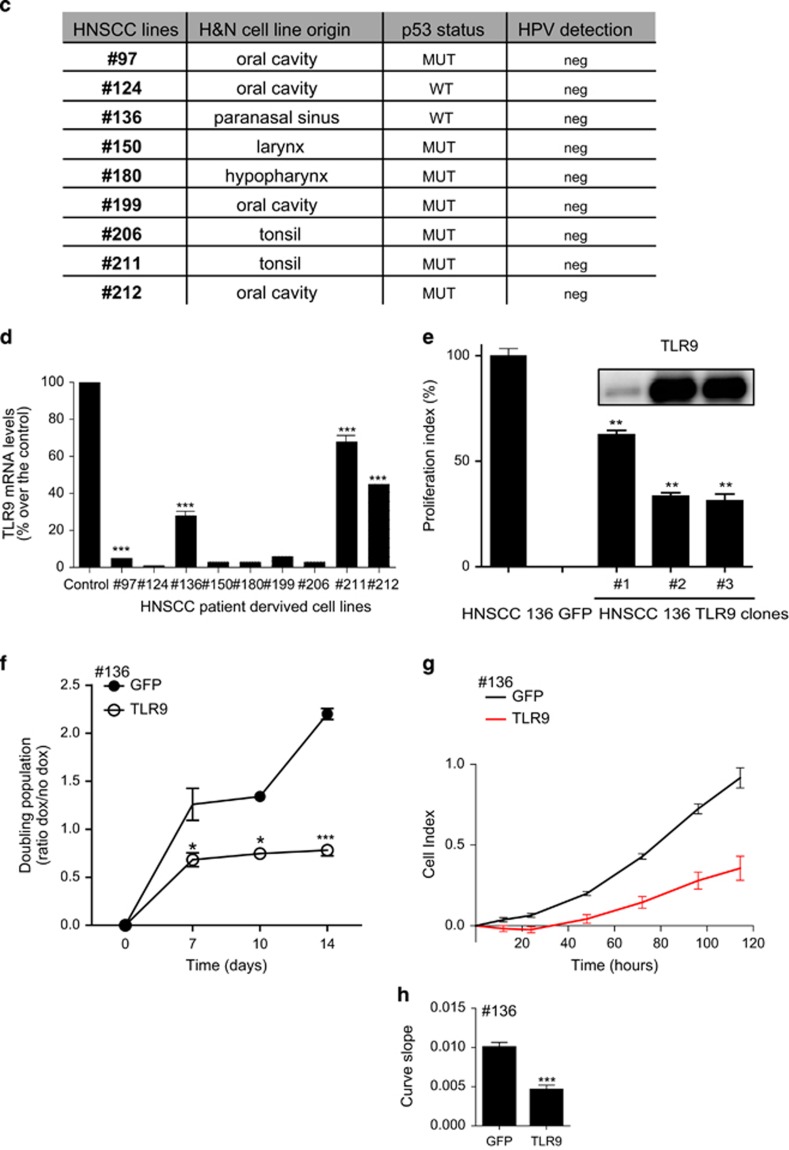
For figure caption please see next page.

We and others have reported the loss of TLR9 expression in several viral induced cancers.^21, 22, 23, 24, 25^ Our next aim was to examine if TLR9 levels were also altered in non-viral induced tumors. Tumor tissue biopsies were taken from 20 patients with head and neck cancer (−HPV) from the IdiPAZ Biobank, La Pas University Hospital, Spain. Two blind histological analysis was performed by two independent pathologists and classified (see table in Figure 2a); sections were stained by immunohistochemistry for TLR9 (Figure 2a). As a positive control for TLR9 staining we selected skin tissues with a normal histological profile (Figure 2b). In agreement with the endoplasmic localization of TLR9, basal cells from the normal epidermis showed strong cytoplasmic staining in control tissues (Figures 2a and b). In head and neck cancer patients weak or no TLR9 staining was observed (Figures 2a and b). We also analyzed normal areas in the patient tissue for TLR9 expression and observed that in most cases TLR9 expression was also reduced (data not shown). Furthermore, in cell lines derived from an independent set of head and neck cancer patients.^26^ TLR9 mRNA levels were reduced (Figures 2c and d). We next determined whether restoring TLR9 expression would have an effect on cell growth in head and neck cancer cells. As constant TLR9 exogenous expression in certain experiments caused cell death, we decided to generate an inducible TLR9 expression vector under the control of tetracycline (doxycycline) using the lentiviral vector pLVUT'.^27^ TLR9 expression was optimal between 3–5 days post induction with doxycycline in HEK293 cells (data not shown). We chose the head and neck cancer cell lines (HNSCC) 124 and 136 to generate TLR9-expressing clones. Induction of TLR9 in the 124 cell line led to immediate cell death. We were able to obtain clones for HNSCC 136 in which we observed optimal TLR9 expression 5 days post induction with doxycycline (Figure 2e and Supplementary Figure 1A,B). We observed calreticulin (an ER marker in red) and TLR9 (in green) were localized and expressed at similar levels HNSCC 136 cells compared with a TLR9 naturally expressing cell line (C33A) (Supplementary Figure 1A,B). We also found that TLR9 overexpression in the HNSCC 136 cell line led to a decrease in cell proliferation compared with the green fluorescent protein (GFP) vector control (Figure 2f). These results show that TLR9 is also downregulated in non-viral induced HNSCC and its re-expression influences cell growth.

### TLR9 expression and not TLR7 or MyD88 affects cell growth

**Figure 3:**
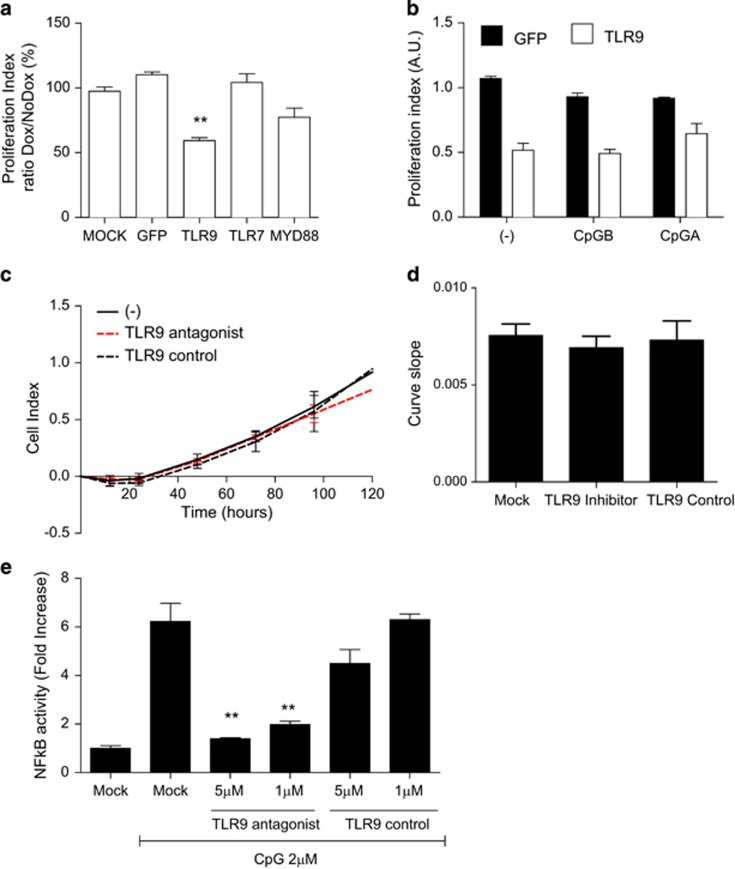
The effect of TLR9 on cell proliferation is not affected by TLR9 engagement. HNSCC 136 were stably transduced with PLVUT'-GFP, PLVUT'-TLR7, PLVUT'-MyD88 and PLVUT'-TLR9 (tet-ON system). Half of the cells were treated with doxycycline during all the experiments. (**a**) After 3 days cells treated with doxycycline or not were seeded and a MTT assay was performed. The proliferation index was calculated as the ratio of the formazan concentration given by the doxycycline condition divided by the one of the no doxycycline condition. (**b**) After 3 days cells treated with doxycycline or not were seeded, stimulated after 24 h for 2 days with CpG A or CpGB, then a MTT assay was performed. The proliferation index was calculated as the ratio of the formazan concentration given by the doxycycline condition divided by the one of the no doxycycline treatment. (**c**, **d**) Cell impendance±TLR9 inhibitor or control. HNSCC 136 TLR9 treated 3 days with doxycycline were plated in Xcelligence 96-well E-16 plate. Doxycycline were added every 2 days and TLR9 inhibitor or control (5μm) at day 1. Impedance was measured every 15 min for 7 days. Curve slopes are presented for each condition. (**e**) TLR9 stably expressing ELAM-luc reporter cells was used to test the TLR9 antagonist. Data are representative of three independent experiments performed in triplicate. The mean±s.e.m. are shown. **P<0.001, based on an unpaired Student's *t*-test.

TLR9 and TLR7 share the same adapter protein MyD88 in their signaling pathway in order to induce immune gene activation.^28^ We therefore tested if MyD88 or TLR7 would also alter cell growth. We observed that neither TLR7 nor MyD88 affected cell growth (Figure 3a). However, using the same inducible system, we observed that in the presence of doxycycline, MyD88-, TLR9- or TLR7-overexpressing cells that were stimulated with CpG and R848, respectively, were able to induce the transient expression of the nuclear factor-κB reporter gene (Supplementary figure 1C,D). Furthermore, the addition of TLR9 ligands CpG oligo type A (CpG 2216) or type B (CpG 2006) (nuclear factor-κB activation) did not influence further the inhibition of cell growth (Figure 3b). We next hypothesized that the anti-proliferative response was ligand mediated and an endogenous DAMP released due to TLR9 expression. The DAMP released would then activate TLR9 to block cell growth. To test this hypothesis, we treated stable TLR9- or GFP-expressing cells with increasing concentration of different cell death inducers, that is, cisplatin, H_2_O_2_ and doxorubicin. No enhanced defect in cell growth was observed as measured by MTT assays (data not shown). Addition of a TLR9 ligand antagonist to block potential TLR9 self-ligands did not alter the effects seen on cell growth (Figures 3c and d) but did inhibit TLR9 CpG activation of the nuclear factor-κB reporter gene (Figure 3e). Therefore, we concluded that TLR9 expression *per se* inhibited cell growth.

### Colony formation and transformation of head and neck cancer cells are altered by TLR9 expression

**Figure 4:**
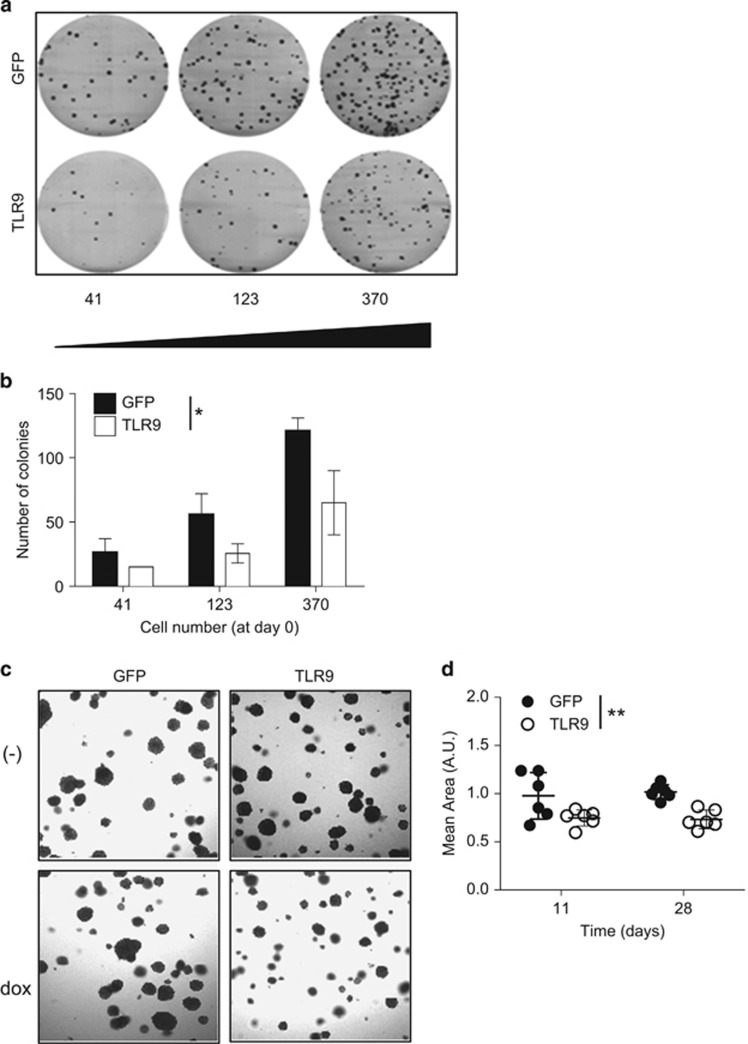
The role of TLR9 on colony formation and transformation. (**a**) HNSCC 136 cells stably transduced with PLVUT'-GFP and PLVUT'-TLR9 and were induced with doxycycline. After 3 days the cells were seeded (41, 123 or 370 cells per well) and 3 weeks after a crystal violet coloration was performed. (**b**) The number of colonies for the HNSCC 136 PLVUT'-GFP (white bars) and the HNSCC 136 PLVUT'-TLR9 (black bars) were counted and reported as noted in the chart. (**c**) PLVUT'-GFP (left panel) and PLVUT'-TLR9 (right panel) HNSCC 136 cells were either induced with doxycycline (lower panel) or left untreated (upper panel). Cells were included in agar and pictures were taken 11 days post inclusion. (**d**) The mean area of the colonies doxycycline+were counted with the software image from the pictures taken 11 and 28 days post inclusion. The mean area of colonies from cells that expressed GFP are in black and TLR9 are in white. Data are representative of three independent experiments performed in triplicate. The mean±s.e.m. are shown. **P<0.001 and *P*<0.01 based on an unpaired Student's *t*-test.

Clonogenic assay or colony-formation assay is an *in vitro* cell survival assay based on the ability of a single cell to grow into a colony. We observed that overexpressing TLR9 abrogated the ability of 136 cells to form colonies (Figures 4a and b). Anchorage independent growth of cells in soft agar is one of the hallmarks of cellular transformation and uncontrolled cell growth, with normal cells typically not capable of growth in semisolid matrices. We tested the ability of our 136 head and neck cancer-derived cells to growth under soft agar (Figures 4c and d). We observed that these cells could grow under soft agar. However, the induction of TLR9 expression led to smaller colony growth under soft agar. Our above findings demonstrate that TLR9 expression alone decreases cancer cell proliferation and colony formation as well as inhibit the events that promote transformation.

### TLR9-expressing cells activate an IFN signature

**Figure 5:**
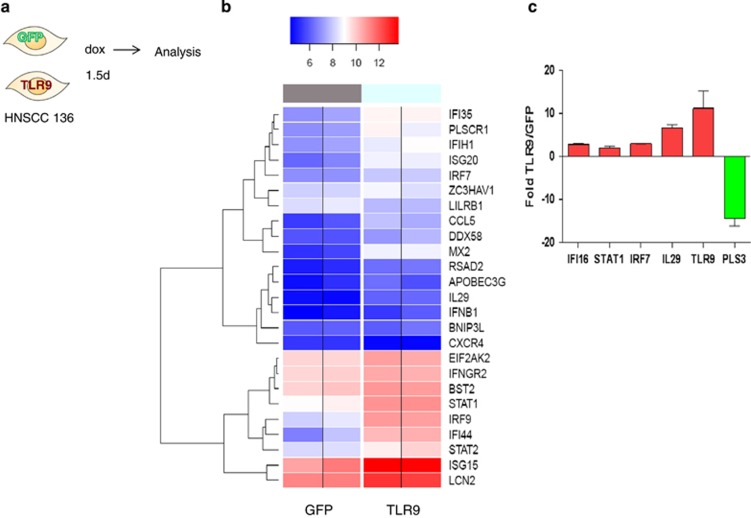
Transcriptome changes induced by TLR9 re-expression. (**a**) Schematic representation of the experiment. HNSCC 136 PLVUT'-GFP and HNSCC 136 PLVUT'-TLR9 were induced for 36 h with doxycycline. The mRNA was extracted and analyzed by microarray. (**b**) 'the true log2 of the quantile normalized expression signals in four samples belonging to two groups, GFP & TLR9 (No ratio and no scaling). In order to enhance data reading, color separation (blue=low and red=high) was based on the 50th theoretical percentile of the expression matrix range' (**c**) cDNA from HNSCC 136 PLVUT'-GFP and HNSCC 136 PLVUT'-TLR9 induced for 36 h was subjected to qPCR to determine the level of expression of IFI16, STAT1, IRF7, IL-29, TLR9 and PLS3. The ratio of expression TLR9 divided by GFP was calculated and plotted on the chart. Data have been disposed on the GEO: number being generated.

We wanted to determine the molecular processes that might be involved in TLR9 control of cell growth and the events that lead to transformation. We therefore performed microarray-based whole-genome expression profiling analysis on HNSCC 136 cells with inducible TLR9 or GFP control at 36 h post induction with doxycycline (Figure 5a). A microarray-based whole-genome expression profiling analysis on HNSCC 136 cells with inducible TLR9 or GFP control at 36 h post induction with doxycycline (Figure 5a). Using the BRB-ArrayTools Version: 4.2.1 program, we noted that at 36 h post TLR9 induction, 415 genes were significantly up- or downregulated ranging from 2 to 14.6 absolute fold differences (*P*-value<0.001; false discovery rate <0.05; data not shown). From Gene ontology annotation implemented in the Database for Annotation, Visualization and Integrated Discovery program (https://david.ncifcrf.gov/), we selected genes that were upregulated and grouped under the ‘response to virus' and ‘negative effect on cell proliferation' at the 5% false discovery rate level, and used the analysis of variance model to visualize the differentially deregulated genes into a heat map analysis (Figure 5b and Supplementary Figure 2). We selected some IFN related and others important genes for validation by quantitative PCR (qPCR; Figure 5c).

**Figure 6:**
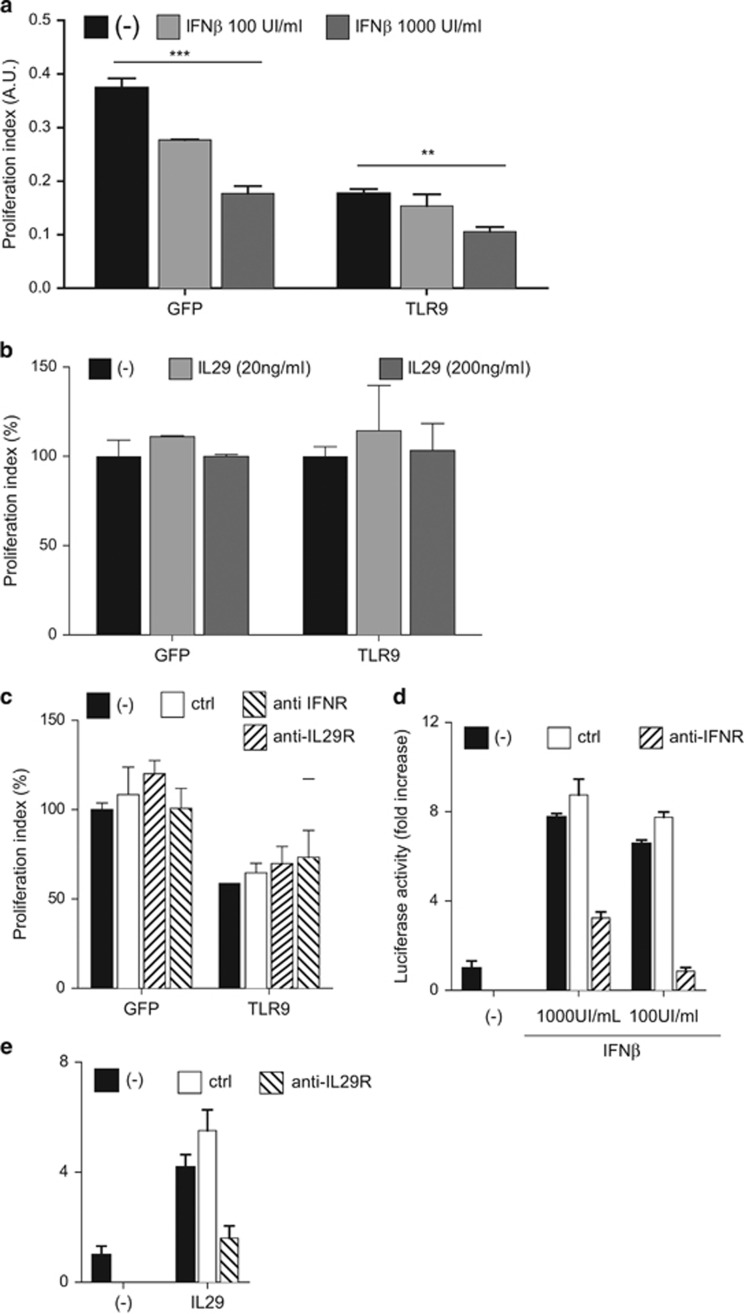
Type I and type III IFN are not involved in TLR9-mediated effects. (**a**) HNSCC 136 PLVUT'-GFP or TLR9 were treated 3 days with doxycycline. Cells were then plated at 10^4^ cells/ml in 96-well plate in presence or not of recombinant IFN-β (100–1000 UI/ml) (**a**) or (**b**) IL-29 (20–200 ng/ml). Doxycyclinee was added every 2 days and proliferation was measured by MTT assay over 4 days. Data are presented as proliferation index of dox conditions only as a percentage over the medium conditioned. (**c**) HNSCC 136 PLVUT -GFP or TLR9 were treated 3 days with doxycycline cells were then plated at 10^4^ cells/ml in 96-well plate in presence or not of ctrl antibodies (20 μg/ml), anti- IL-29 and anti-IL-10Rb (20 μg/ml) or and IFN-β (1000 or 100 UI/ml) and anti-IFNAR2 (5 μg/ml). Doxocycline was added every 2 days and proliferation was measured by MTT assay over 4 days. Data are presented as proliferation index of dox conditions only as a percentage over the ctrl antibody condition. (**d**,**e**) Activation of HEK293-Tcells stably transfected ISRE reporter was measured by luciferase assay. 293T cells were plated in 96-well plate at 10^5^ cell/ml. 24 h later the control, anti-IFN-β (10 g/ml), anti-IFNAR2 (5 μg/ml) or anti-IL-29 (20 μg/ml) were added for 1 h then IFN-β (100 or 1000 UI/ml), IL-29 (200ng/ml) or medium were added for an additional 24 h. Data are represented as fold over the medium conditions. Data are representative of three independent experiments performed in triplicate. The mean±s.e.m. are shown. ***P<0.0001 and **P<0.001 based on an unpaired Student's *t*-test.

*IFIH1, IL-29, IRF9, IFNB1* and *IRF7* were upregulated under the responses to virus classification. IFN genes *IL-29, IFNB1* and *STAT1* were upregulated and also grouped under negative effect on proliferation. A panel of selected IFN-regulated genes as well as control genes TLR9 and pLS3 (a gene that was downregulated) were validated by qPCR (Figure 5c). We then postulated that autocrine production of IFN-I or IL-29 by TLR9-expressing HNSCC 136 cells may contribute to the decrease in cell proliferation. We examined if type I (IFNα/β) or type III IFN (IL-29) *per se* affected cell growth. IFN-β decreased cell growth in 136 cells that expressed TLR9 or GFP (Figure 6a); however, addition of IL-29 did not (Figure 6b). Moreover, neither IL-29R nor type I IFNR-blocking antibodies altered the inhibitory effect on cell proliferation by TLR9 on 136 cells (Figures 6c–e). Although TLR9 overexpression does induce an IFN-type signature; our data demonstrate that IFN or IL-29 were not responsible for cell growth inhibition.

### TLR9 induces a slowdown of the S-phase in head and neck cancer cells mediated by p16^INK4a^

**Figure 7:**
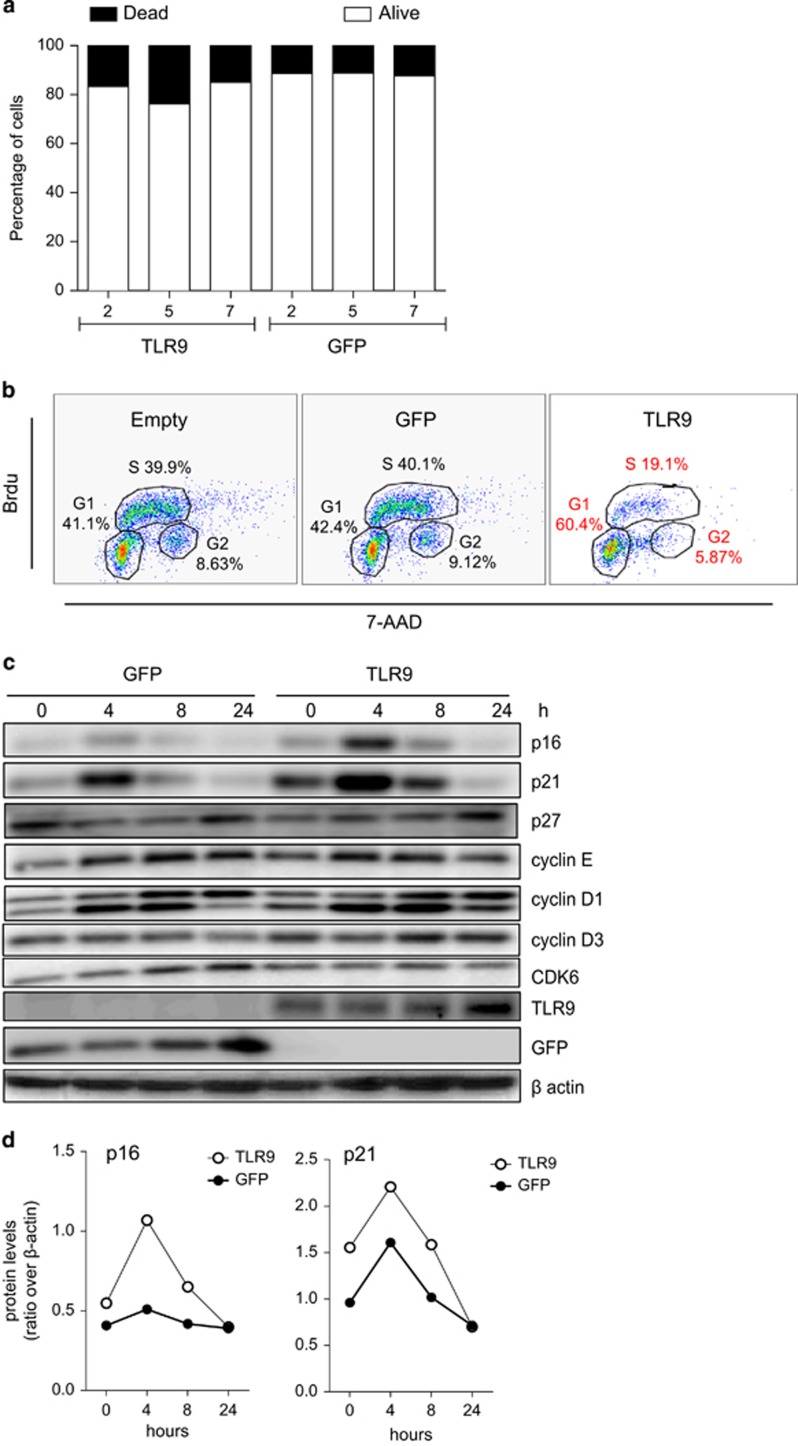
TLR9 expression affected the cell cycle but not apoptosis. (**a**) Caski cells stably transduced with GFP (left panel) or TLR9 (right panel) encoded pbabe were stained with Annexin V and propidium iodide to determine percentage of apoptotic and necrotic cells. (**b**) HNSCC 136 cells were stably transduced with pbabe (left panel), pbabe -GFP (middle panel) or pbabe-TLR9 (right panel). Cells were pulsed with BrdU for 20 min and cell cycle analysis was performed after BrdU and 7-AAD staining by flow cytometry. (**c**) HNSCC 136 PLVUT'-GFP (left panel) and HNSCC 136 PLVUT'-TLR9 (right panel) were induced with doxycycline and serum deprivated for 2 days. Cells lysate were collected 0, 4, 8 or 24 h after serum addition. Expression of cell cycle proteins were analyzed by western blotting. (**d**) Densitometry analysis of the western blot band from (**c**).

**Figure 8:**
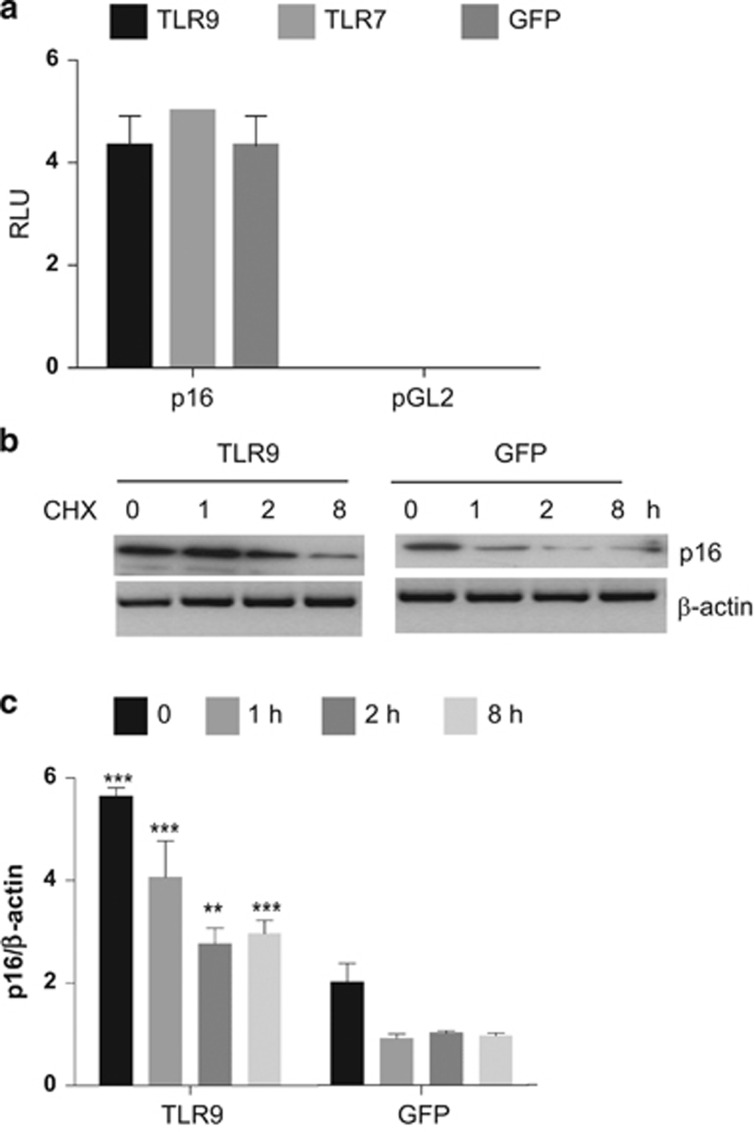
TLR9 increases p16 protein stability. (**a**) HEK293T cells were co-transfected with the p16 promoter linked to the luciferase gene or pGL2 and TLR9, TLR7 or GFP pCDNA plasmids. After 48 h of transfection, cells were lysed and luciferase activity was measured. (**b**) Ability of TLR9 to alter p16 protein levels. The indicated HNSCC cells were cultured in a medium containing cycloheximide. The levels of p16 and β-actin (loading control) were determined by immunoblotting (**c**) and the amount of p16 and β-tubulin was quantified by Multligauge imager in three independent experiments.

We next wanted to determine cell growth inhibition observed in TLR9-expressing cells also promoted cell death by late apoptotic or necrotic process using PI or 7-AAD inclusion. TLR9-expressing cells did not induce significant cell death (Figure 7a) as shown by PI or 7-ADD staining. As TLRs have been shown to alter cell proliferation^11, 14, 19^ we examined if TLR9 affected the cell cycle. Therefore, the cell cycle length in the head and neck cancer cell line 136 post TLR9 induction was analyzed after cell synchronization using thymidine double blockage. Successive labeling BrdU for 15 min demonstrated that TLR9 induced an accumulation of the G1 phase and lengthening of S-phase (Figure 7b). Reported by Chien W *et al.*^29^ was the ability of p16^INK4a^ in human cancer cells to lengthen the S-phase along with the accumulation G1 phase in the cell cycle. Synchronized cells showed that p16^INK4a^ levels were increased in TLR9-expressing cells (Figures 7c and d). Recently Yu *et al.* showed that DNA damage in mammalian cells can signal cell-autonomously to induce endogenous IFN-β in an IRF3-dependent manner. The expression of endogenous IFN-stimulated genes further activated the p53–p21 axis and increased the levels of p16^INK4a^ concurrent with robustly promoting cell senescence *in vitro*.^30^ Although TLR9 expression did lead to induce IFN stimulatory factors (Figure 5b) it did not lead to significant changes in p16^INK4a^ mRNA levels (data not shown) or promoter activity (Figure 8a). As p16^INK4a^ levels were already elevated in TLR9-expressing cells at time point 0, we hypothesized that the protein stability of p16^INK4a^ may be affected owing to TLR9 expression. To test our hypothesis, 136 cells were transduced with TLR9 or the GFP control and were cultured in a medium containing cycloheximide to inhibit protein synthesis. p16^INK4a^ half-life was determined by immunoblotting. In TLR9-expressing cells we observed an increase in p16^INK4a^ stability compared with the GFP control (Figures 8b and c). These findings demonstrated the mechanism by which TLR9 expression can mediate cell growth slowdown by increasing p16^INK4a^ stability. Our data highlight a central role for TLR9 in cancer development, therapeutic strategies to reactive its expression should be targeted.

## Discussion

The major role of TLRs is to defend the host against pathogens via pathogen-associated molecular pattern recognition, which triggers the innate immune response. We and others share evidences suggesting that loss of TLR9 expression may lead to a poor immune response in viral related cancers. Wu *et al.*,^31^ showed that a SNP on the TLR9 promoter at position −1237, allows increased TLR9 expression that correlated to earlier HBeAg seroconversion. The −1237 was also identified by our group as being the site of interaction in which nuclear factor-κBp65/ERα suppressed TLR9 transcription and thus IFN production.^20^ Earlier findings from our team revealed that Epstein Barr virus infection of primary B cells led to a decrease in TLR9 mRNA levels, which became increasingly distinct upon immortalization, signifying that the reduction in TLR9 expression may be linked to cellular transformation (19). Indeed, TLRs also has a role in tissue repair, cell proliferation, apoptosis and angiogenesis.^10, 11, 14, 16, 17^ The later activities link TLR signaling to cancer. Although there are evidences that demonstrate specifically that TLR9 is altered in cancer cells,^19, 32, 33^ proving their role in carcinogenesis remains changeling. A recent study by Zambirins *et al.*,^13^ suggest that TLR9 ligation in pancreatic stellate cells promoted tumorigenesis. Also in a model of hepatocellular carcinoma; hypoxia induced HMGB1 with released mitochondrial DNA lead to the activation of TLR9-mediated tumor growth.^34^ On the contrary, TLR9 is downregulated in a several viral and non-induced cancers and that different oncoviruses.^19, 23, 24, 25, 33, 35, 36, 37^

Here we have shown that TLR9 expression was reduced in several HNSCC derived from patients. These results were corroborated in tissue biopsies from an independent cohort of head and neck cancer patients. Our new data have showed that the loss of TLR9 expression may also disable its ability to control the cell cycle and events that may control transformation. These results highlight the association of TLR9 in cell cycle control in cancer cells. We show for the first time that TLR9 *per se* in head and neck cancer cells was able to slowdown the cell cycle during the S-phase. p16^INK4a^ can act as a tumor suppressor that is implicated in the prevention of cancers, notably melanoma, oropharyngeal squamous cell carcinoma, cervical cancer and esophageal cancer.^29^ Chien *et al.*,^29^ described that p16INK4a overexpression led to an extended S-phase in cancer cells that contained WTp53. We showed that HNSCC 136 cells, which contained p53WT, which TLR9 expression increased p16^INK4a^, which coincided with an extension in the S-phase. The S-phase has an essential role in that any problems with DNA replication trigger a 'checkpoint'—we hypothesize that TLR9 expression leads to signaling events that puts the S-phase on hold until the problem is resolved. It has recently been shown that TLR9 expression is strongly activated via p53 in primary human blood lymphocytes and alveolar macrophages upon exposure to different types of DNA-damaging insults.^27^ We speculated that the inhibitory role of TLR9 on cellular proliferation was induced by DAMPs. However, in our experimental setting we were unable to demonstrate that DAMPs released owing to cell death were responsible for TLR9-mediated slowdown in cell growth. Our collaborative work with Pacini *et al.*^19^ also showed that re-expression of TLR9 in HPV38 E6/E7 HFK resulted in a strong accumulation of the cell cycle inhibitors p21^WAF1^ and p27^Kip1^ and a clear decrease in cellular proliferation. The increase of p21^WAF1^ due to TLR9 re-expression was also observed in our model of head and neck cancer (also upregulated in our microarray data: Series record GSE78858). Unlike the study from Pacini *et al.*^19^ we did not observe increased p-p38 or as our previous work has shown p27^Kip1^ (data not shown). In our experiments we observed that TLR9-expressing cells increased p16^INK4a^ protein, which has been shown to positively control the expression of p21(WAF1).^38^ We still have to elucidate how TLR9 re-expression allows for p16^INK4a^ stability. p14ARF has been shown to stabilize p16^INK4a^. p14ARF regulates the stability of p16^INK4a^ protein via REGγ-dependent proteasome degradation. Kobayashi *et al.,*^39^ therefore, it would be interesting to determine whether TLR9 re-expression in our model can induce p14ARF. We cannot exclude that certain IFN regulatory transcription factors may also influence the stability of p16^INK4a^.^30, 40^ In conclusion, in addition to escaping immune recognition, we have shown that the deregulation of TLR9 in viral and non-viral induced cancer may also favor carcinogenesis. Most importantly, our study highlights a novel function of TLR9 in negatively regulated cellular proliferation by increasing the stability of p16^INK4a^.

## Materials and Methods

### Expression plasmids

The human TLR9 sequence was previously cloned and described.^41^ Human TLR9 and GFP were cloned in the retroviral vector pBabe-puro.^42^ The pLVUT is a lentiviral vector expressing GFP downstream from the ubiquitin promoter in a doxycycline-inducible manner.^27^ PLVUT' was created from pLVUT to generate a unique EcoRI site downstream of the GFP gene. The EcoRI site at position 6235 was removed by digestion with BstB1. Human TLR9, MyD88 and TLR7 cDNA were cloned into lentiviral pLVUT' vector. Tetracyclin-inducible PLVUT' vector, TLR9, MyD88 and TLR7 were generated by the ISP (Innate Sensors Platform). The pLXSN, HPV16E6E7, pGL3-NF-kB luciferase, pGL3-ELAM luciferase, pGL4-TK and Renilla constructs have been previously described.^20, 36^ The p16^INK4a^-luciferase construct was cloned as described by.^43^

### Cell lines

HEK293 and cervical cancer-derived cell lines, SiHa and Caski were obtained from American Type Culture Collection. HNSCC cell lines have been previously described.^44^ Cells were maintained in Dulbecco's Modified Eagle Medium supplemented with 10% fetal bovine serum and 10μg/ml ciprofloxacin. Primary human foreskin and embryonic keratinocytes were isolated and grown together with NIH 3T3 feeder cells in FAD medium (Cascade) as previously described,^36^ or when in the absence of feeder cells, keratinocytes were grown in EPI-LIFE medium (Cascade) supplemented with growth factors (Cambrex, New Jersey, NJ, USA) and 10 ng/ml human epidermal growth factor (R&D Systems, Lille, France). Cells were cultured at 37 °C with 5% CO2. The Caski and HNSCC 136 PLVUT'-GFP and TLR9 were generated after lentiviral transduction and cloned by limited dilution. Ten clones for each cell line positive for WPRE were selected and amplified.

### Lentiviral and retroviral infections

Retroviral infections have been previously described.^36^ Infected cells were selected with puromycin (1 μg/ml) for 3 days (corresponding to 100% of killing of uninfected cells). Lentiviral particles were produced by the 'plateau technique analyse genetique et vectorologie' (SFR Biosciences UMS3444/US8, Lyon, France). Lentiviral infections were done accordingly to the protocol of the ISP.

### RNA extraction, reverse transcriptase-PCR and qPCR

RNA was extracted using Nucleospin RNA/protein kit following the manufacturer protocol (Macherey-Nagel, Germany). Reverse transcriptase reaction was performed using 500–1000ng of RNA. For qPCR, complementary DNA were diluted 1/20 for quantitative PCR (qPCR) reactions using Mesa green qPCR Master Mix (Eurogentec, Angers, France). PCR was conducted using the Mx 3000P real-time PCR system (Stratagene, La Jolla, CA, USA). Two sets of PCR assays were conducted for each sample, the TLR9 and β2-microglobulin primers have been described.^35, 45^ Amplification specificity was assessed for each sample by melting curve analysis, and the size of the amplicon checked by electrophoresis (data not shown). Relative quantification was performed using standard curve analysis. TLR9 mRNA levels were normalized to β2-microglobulin mRNA levels and are presented as a ratio of gene copy number per 100 copies of β2-microglobulin in arbitrary units.

### Functional analysis

Synthetic phosphodiester oligodeoxynucleotides (CpG 2006 and GpC 2006) were synthesized by InvivoGen (Toulouse, France) and used at indicated concentration. IFN-β was used at 100 or 1000 UI/ml (Avonex, Biogen, Nanterre Cedex, France), anti-IL-29, anti-IFNβ (R&D) and anti-IFNAR2 (PBL, New Jersey, NJ, USA). Cells were stimulated overnight and the response was monitored by luciferase assay or enzyme-linked immunosorbent assay. For luciferase assay transient transfection of the reporter plasmid NF-kB, or ELAM luciferase was performed as previously described.^41^ Enzyme-linked immunosorbent assays were done in accordance to manufacturer's instructions (R&D system).

### Immunoblot analysis

In brief, harvested cells were lysed in mild lysis buffer containing 50 mM Tris-HCl (pH 8.0), 150 mM NaCl, 1% Triton X-100, 1 mM DTT, 0.5 mM and complete protease inhibitor (Roche, Meylan, France). Cellular protein content was determined by the Bradford assay (Bio-Rad, Marnes-la-Coquette, France); used for sodium dodecyl sulphate-polyacryl amide gel and immunoblotting onto a polyvinyl difluoride membrane. After incubation with primary antibodies, proteins were detected with secondary peroxidase-conjugated antibodies (Promega, Madison, WI, USA) and ECL. All the primary antibodies for western blotting were from Cell Signaling but the β actin (MP biomedicals, Santa Ana, CA, USA).

### Proliferation assay

Doubling population assay and clonogenicity assay were previously described.^46^ For MTT assays, cells were plated at 50 000, 100 000 and 150 000 cell/ml in 96-well plate in quadruplicate. After 48 h 10% of 3-(4,5-dimethylthiazol-2-yl)-2,5-diphenyl tetrazolium bromide (5 mg/ml) (Sigma-Aldrich, St Louis, CA, USA) was added for 4 h then supernatants were removed and the cells resuspended in dimethyl sulphoxide. The 550nm absorbance was read on a plate reader. For the flow cytometry proliferation analysis, the cells were either stain with PHK 26 fluorescent dye (Sigma-Aldrich, France) or CellTrace violet Cell proliferation kit (Life technologies, France) following manufacturer recommendations. Cell Proliferation Assay*s*: for each cell type, the indicated number of cells/well was seeded into 100 μl of media in 96X microplates (E-Plate). The attachment, spreading and proliferation of the cells were monitored every 30 min using the RT-CES system. Cell proliferation was monitored for 48–72 h, depending on the experiment. Cell-sensor impedance was expressed as an arbitrary unit called the Cell Index. The rate of cell growth was determined by calculating the slope of the line between two given time points.

### Flow cytometry analysis

For synchronization experiments, 200 000 cells/ml were plated in a six-well dish. The next day thymidine (2 mM final) was added to the cells, 18 h later the cells were washed three times with PBS and complete medium was added for 9 h. Then thymidine (2 mM final) was added for 18 h. The cells were then washed three times in PBS and complete medium containing BrdU was added. For BrdU cell cycle analysis of non-synchronized cells, 100 000 cells per well were plated. The next day BrdU was added to the plate for 20–30 min. The cells were harvested and washed in PBS then fixed in 70% ethanol overnight at 4 °C. The cells were treated with 3N HCl that was neutralized with 0.1 M Na2B4O7 pH 8.5. The cells were then blocked and stained with an anti-BrdU (Biolegend, London, UK) and 7-AAD (Life Technologies). For apoptosis experiment, Apoptosis detection kit I (Becton Dickinson, Le Pont de Claix, France) was used following manufacturer protocol. For TLR9 intracellular staining, one million cells were fixed with 2% paraformaldehyde and permeabilized in PBS containing 0.25% saponin. After blocking the cells were stained with a rat anti-human TLR9 (eBioscience, Paris, France) and with a secondary goat anti-rat Alexa 633. The staining was analyzed on a BD LSRII using the software Diva and FlowJo (Treestar, Ashland, OR, USA).

### Histology

IdiPAZ biobank, La Pas University Hospital, Spain. TLR9 staining was performed as previously described^36^ and scoring was performed by pathologist Alexandra- Traverse Glehen (Department Laboratoire d'Anatomie et cytologie pathologiques, Hopital Lyon Sud, France).

### Soft agar

TLR9 or GFP HNSCC 136 cells were plated (150 000 cell/well) in six-well dish and induced in presence or not of doxycycline for 48 h. Cells were then trypsined and counted to include in soft agar assay. Soft agar assay consisting of a 2.5  ml lower layer of 0.75% agar in double-strength Dulbecco's Modified Eagle Medium supplemented with 7.5 g/l NaHCO3 and 10% fetal bovine serum was placed in a six-well plated and permitted to solidify at room temperature. Cells to be tested for colony formation were suspended (22 500 cell/well) in a plating layer of 0.45% agar in double-strength Dulbecco's Modified Eagle Medium containing 10% fetal bovine serum in presence or not of doxycycline. About 100 μl of double-strength Dulbecco's Modified Eagle Medium containing fetal bovine serum, and doxycycline if indicated, was added to soft agar every 3 days. Pictures were taken 11 days post inclusion.

### Isolation of RNA from HNSCC 136 cells

HNSCC 136 cells transduced with pLVUT' TLR9 or GFP where plated with the same number of cells and cells were harvested and RNA extracted post as mentioned above post 36 h treatment with doxycyline. RNA concentration and purity were evaluated with the Nanodrop (Thermo Scientific, Illkirch, France). RNA integrity and quantification were characterized by measuring the 28 s/18 s rRNA ratio and RIN (RNA Integrity Number) using the Agilent 2100 bioanalyzer instrument and the RNA 6000 Nano kit. The RIN software classifies the integrity of eukaryotic total RNAs on a scale of 1–10, from most to least degraded.

### Microarray-based whole-genome expression profiling and data analysis

Genome-wide gene expression profiling analysis was performed on Illumina HumanHT-12 v4 Expression BeadChips, providing a coverage of >24 000 annotated genes (48 783 probes corresponding to 1–3 probes per gene) including well-characterized genes and splice variants. Candidate probe sequences included on the HumanHT-12 v4 Expression BeadChip derive from the National Center for 8 Biotechnology Information Reference Sequence RefSeq (Build 36.2, Rel22) and the UniGene (Build 199) databases. Using the Illumina TotalPrep RNA Amplification Kit (Ambion, Thermofisher, Illkirch, France), 500 ng of extracted RNAs were converted to complementary DNAs and subsequent biotin labeled single-stranded cRNAs. The distribution of homogeneous *in vitro* transcription products (cRNAs) was checked with the Agilent 2100 bioanalyzer instrument and the RNA 6000 Nano kit. In total, 750 ng of biotin labeled cRNAs of the four (biological triplicates) samples were hybridized overnight to four HumanHT-12 Expression BeadChips. Subsequent steps included washing, streptavadin-Cy3 staining and scanning of the arrays on an Illumina BeadArray Reader. Fluorescence emission by Cy3 was quantitatively detected for downstream analysis. The Illumina Genome Studio V2010.2 was used to obtain the signal values (AVG-Signal), with no normalization and no background subtraction. Data quality controls were performed using internal controls present on the HumanHT-12 beadchip and were visualized as a control summary plot and for each sample as noise-to-signal ratios calculated by P95/P05 signal intensities. All samples had P95/P05 >10, defined as sample quality threshold (data not shown).

Differential expression analysis was performed using BRB-ArrayTools software v4.2 developed by Dr Richard Simon and BRB-ArrayTools Development Team.^47^ The raw signal intensities of all samples were log-transformed and quantile normalized with background subtraction with the exclusion of any probe showing excess dispersion (defined by >85% of individual probe values differing from the median by >1.5-fold). Class comparison for Microarray Analysis using the *t*-test method was performed for identification of differentially expressed probes. Probes with a *P*-value of <0.001, with a minimum of twofold change and a false discovery rate of <0.05 were considered significantly differentially expressed. The Database for Annotation, Visualization and Integrated Discovery v 6.7 was used for classification of the differentially expressed genes.^48^

### Heat map generation

Pre-treatment of data and graphics were carried out with the R statistical langage–Version 3.2.2. In brief, we used the R limma program to perform background correction & quantile normalization.^49^ After normalization, the intensities were transformed into log2 and the control probes were removed.
