# Supplementary material for: TLR9 re-expression in cancer cells extends the S-phase and stabilizes p16INK4a protein expression
Source: Oncogenesis. 2016 Jul 25;5(7):e244–. doi: 10.1038/oncsis.2016.49 (PMC4972902; doi:10.1038/oncsis.2016.49)
Supplement: Supplementary Figure 1 [file oncsis201649x1.pdf]

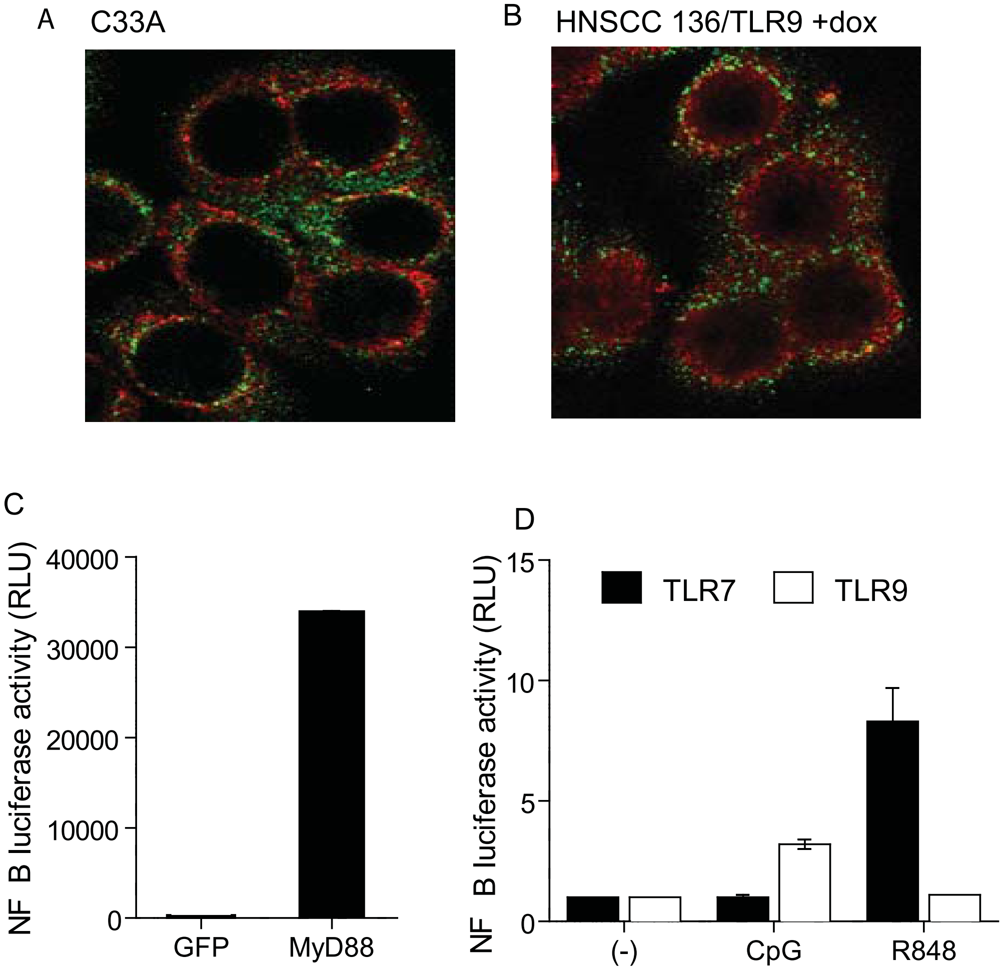

Figure 1: (A) TLR9 expression was analyzed in C33A cells by confocal microscopy.
TLR9 (green) and calreculin (red) staining.
(B) TLR9 expression was analyzed in HNSCC-136 –TLR9 cells by confocal microscopy.
TLR9 (green) and calreculin (red) staining. (C) HEK293 cells were co-transfected with pbabe expressing (C) MyD88, (D) TLR9 or TLR7 with the NF-κB luciferase reporter construct and the TK-Renilla reporter.
Eighteen hours post transfection cells were stimulated with 3uM of CpG-B or by R848.
Cells were harvested and luciferase activity measured and normalized by the Renilla.
