# Supplementary material for: TLR9 re-expression in cancer cells extends the S-phase and stabilizes p16INK4a protein expression
Source: Oncogenesis. 2016 Jul 25;5(7):e244–. doi: 10.1038/oncsis.2016.49 (PMC4972902; doi:10.1038/oncsis.2016.49)
Supplement: Supplementary Figure 2 [file oncsis201649x2.pdf]

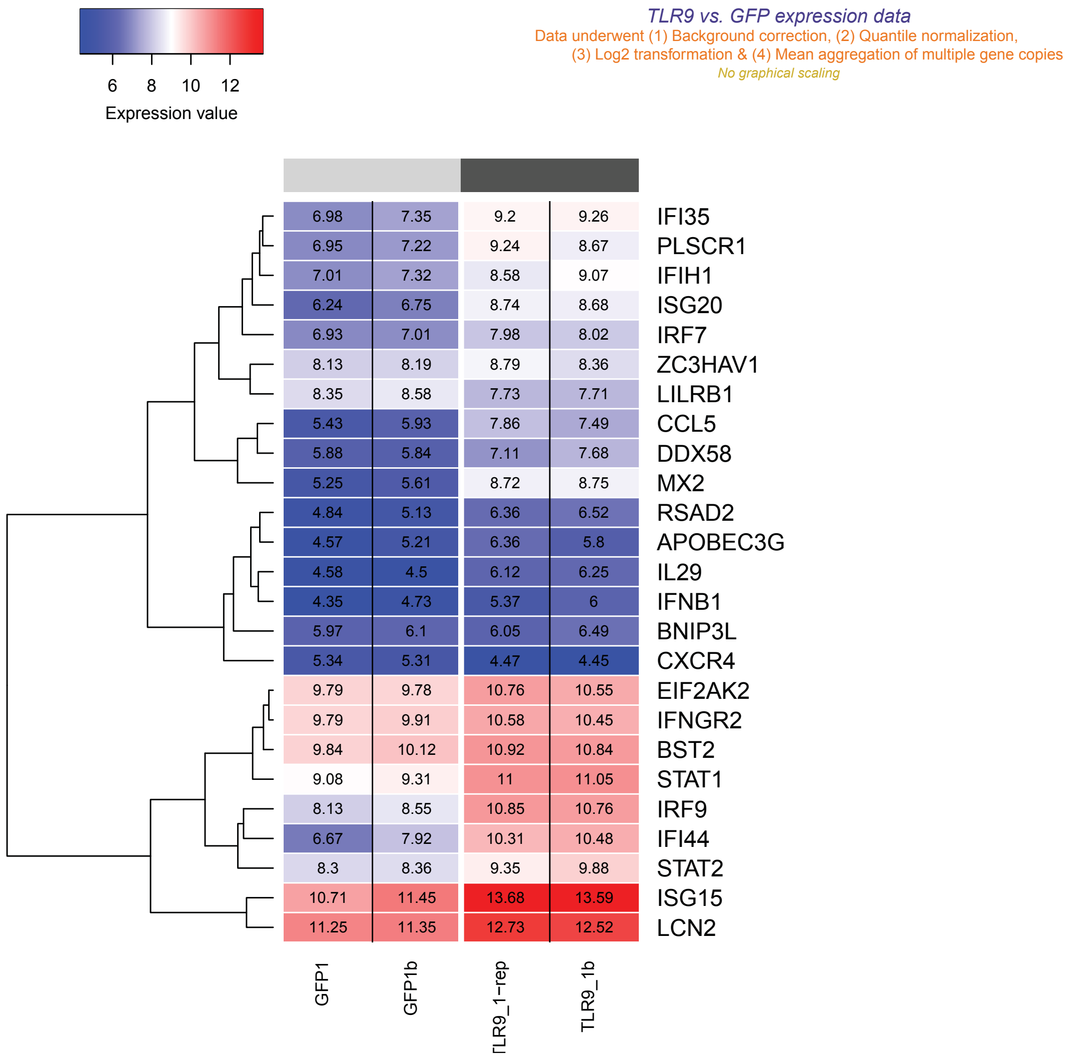

Supplementary Figure 2

Figure 2: The true log2 of the quantile normalized expression signals in 4 samples belonging to 2 groups, GFP & TLR9 (No ratio & no scaling). In order to enhance data reading, color separation (blue = low & red = high) was based on the 50th theoretical percentile of the expression matrix. Here fold values are shown.
